# Apollo: An Event-Based Direct Detector for MicroED

**DOI:** 10.1063/4.0001056

**Published:** 2025-10-27

**Authors:** Michael S Spilman

**Affiliations:** Direct Electron LP, San Diego, CA, USA

## Abstract

The widespread establishment of cryo-electron microscopy (cryo-EM) facilities equipped with electron-counting direct electron detectors (DEDs) offers new opportunities for their application in microED experiments. Electron counting offers significant improvements in sensitivity necessary for detecting high-resolution diffraction spots. The limited linear range of most frame-based electron-counting DEDs makes them prone to coincidence loss from the higher intensity of low-resolution reflections. Recent advancements in event-based electron counting (EBEC) technology, as demonstrated by the Direct Electron Apollo detector, significantly reduce coincidence loss and allows it to capture stronger reflections without saturating (Figure 1). Apollo uses a novel event counting monolithic active pixel (MAPS) sensor with on-chip CDS and edge computing to count, upsample and sum the data within the camera hardware enabling much higher counting rates. Initial small-molecule microED experiments using the Apollo detector of sodium glutamate and histidine resolved to a remarkable 0.5 Å (Figure 2). These high-resolution datasets were acquired approximately ten times faster than those obtained with traditional frame-based MAPS detectors. Apollo's improved sensitivity and acquisition speed streamline experimental workflows and support high-throughput crystallographic screening, significantly expanding the practical applications of MicroED in structural biology and pharmaceutical research.

